# Visual function of drivers and its relationship to road traffic accidents in Urban Africa

**DOI:** 10.1186/2193-1801-3-47

**Published:** 2014-01-24

**Authors:** Godswill Pepple, Adedayo Adio

**Affiliations:** Eye clinic, Braithwaite Memorial Hospital, PortHarcourt, Rivers State Nigeria; Department of Ophthalmology, University of Port Harcourt Teaching Hospital, PortHarcourt, Rivers State Nigeria

**Keywords:** Driving vision, Road safety, Road traffic accidents, Port Harcourt, Alcohol intake, Nigeria

## Abstract

**Aim/background:**

Nigeria has one of the highest mortality rates from Road traffic accidents (RTAs). Prevention is a global priority. This study is aimed at acquiring information for effective policy formulation to improve safety on Nigerian roads.

This is a cross sectional descriptive study in which consenting commercial drivers in a Nigerian motor parks were ophthalmically examined after an interview. Data analyzed with EPI-INFO version 6.0 statistical software using Chi square. P value < 0.05 was considered to be significant.

**Results:**

The eyes of 400 commercial drivers were examined. Ages range from 25 to 62 years with mean of 37.8 years (SD ± 9.1) 20% did not undergo any prior driving test and only one third of those who had (n = 120, 30.9%) had a prior eye test. Up to 45.5% (n = 182) had been involved in RTAs with alcohol intoxication and driver fatigue significantly associated. Depressants such as alcohol are commonly used**.** Visual impairment ((p = 0.46, χ ^2^ = 0.3, RR = 0.62), visual field defects (p > 0.05, χ^2^ = 0.78, RR = 1.25) and color vision defects (p = 0.4, χ ^2^ = 0.77, RR = 1.23) were not significantly associated with occurrence of RTAs. However fatigue was found to be a predisposing factor in 28% of those who had RTAs.

**Conclusion:**

One out of every two commercial driver has been involved in an RTA in the past in Nigeria. Fatigue while driving should be avoided. Use of depressants while driving should be penalized. Blood alcohol content (BAC) levels should routinely be measured by road safety personnel in Nigeria. Periodic eye exams should be carried out for all commercial drivers before issuing or renewing licenses to drive and treatment for any ophthalmic conditions discovered enforced.

## Introduction

Good visual acuity (VA) in addition to good stereopsis, normal colour vision, satisfactory eye co-ordination and the ability to adapt to various levels of illumination are essential to a driver in order to avoid road traffic accidents (RTAs) (Nwosu 1989).

The causes of RTAs are multifactorial; poor maintenance of roads and vehicles, absence of appropriate road signs and poor driving skills. In addition, deplorable habits of drivers from inadequate training, inattentiveness, alcoholic intoxication, drug intake, excessive speeding, wrong overtaking, poor knowledge of traffic regulations, and physical disability, an example of which is poor vision ((Road safety Nigeria (homepage on the internet) (Cited 2008 Dec. 10) 2013; Facts: vision and driving homepage on the internet June 2005) which has a higher incidence with advancement in age.

An estimated 1.2 million people are killed in RTAs every year worldwide and as many as 50 million suffer injuries (Road safety Nigeria (homepage on the internet) (Cited 2008 Dec. 10) 2013). The World Health Organization (WHO) estimates that these figures could increase by more than half over the next 20 years unless there is a firm commitment to road safety and accident prevention especially in developing countries where the mortality from RTAs rank one of the highest such as Nigeria (Oyemade 1973). Prevention of RTA is now a global priority by the WHO (World Health Organization 1984), resulting in a decline in majority of industrialized countries. However the is not the case in developing nations (World Health Organization 1983).

RTAs result in depletion in the labor force as young people (aged 15–30 years) belong to the group mostly affected (Asogwa 1980). A study has shown that one out of three stand the risk of getting injured and one out of nine stand the risk of getting killed in this Nigerian population, on a yearly basis from RTA (Ezenwa 1986).

In view of the magnitude and impact of RTAs, the Federal Road Safety Commission (FRSC) was established by the Federal Government of Nigeria vide Decree 45 of 1988 (Federal Republic of Nigeria Official Gazette 1988) as amended by Decree 35 of 1992 but effective 18th February 1988.

The FRSC is one of the national and international bodies which have made recommendations about the visual fitness of drivers as contained in “Guidelines for the National Drivers License Scheme” (Agunloye 1990a) For private motor drivers, visual acuity of at least ^6^/_12_ in the better eye and ^6^/_36_ in the poorer eye while for commercial drivers, the minimum visual acuity would be ^6^/_9_ in the better eye and ^6^/_24_ in the poorer eye with or without glasses. The FRSC also laid down minimum standards for a driving license to be obtained (Federal Republic of Nigeria official Gazette (2004) National Road Traffic Regulations, 79th edn.) These include driving school attendance, possession of a learner’s permit, evidence of having passed a driving test carried out by a Vehicle Inspection Officer (VIO), knowledge of the Highway Code, ability to read all road signs and most importantly passing an eye test. These eye tests are seldom carried out however.

This study on the visual function of commercial motor vehicle drivers is aimed at acquiring information for effective policy formulation on the visual aspects of road safety in Nigeria so as to improve safety on Nigerian roads.

## Methods

A cross-sectional descriptive study approved by the University of Port Harcourt Ethics and Research committee was carried out in Port Harcourt Local Government Area (PHALGA) of Rivers State, Nigeria which has 10 approved motor parks (7 Government motor parks and 3 private motor parks (-referring to larger spaces reserved for commercial vehicles waiting to be filled with passengers without causing obstruction to flow of traffic) with both interstate and intrastate transporters using it. All registered consenting active commercial motor vehicle drivers were included.

Accordingly, 400 of the 410 drivers were interviewed with questionnaires in English by face to face interview eliciting information on personal data, driving history, general medical/social history, ocular history and ocular examination between January and April, 2010.

Distant visual acuity at 6 m with the Snellen’s chart or Illiterate ‘E’ chart at 6 m, near vision assessment at 33 cm with a Sussex vision ^R^ near vision chart, external eye examination including extraocular motility examination, fundoscopy with a direct ophthalmoscope, visual field evaluation with suprathreshold Optifield automated perimeter, applanation tonometry with Perkin,s hand held tonometer and color vision assessment using the Ishihara pseudoisochromatic 24 color plate edition were carried out.

Drivers requiring further evaluation were referred to the eye clinic of University of Port Harcourt Teaching Hospital (UPTH) Port Harcourt.

All data was analyzed with EPI-INFO version 6.0 statistical software using Chi square with the aid of a statistician. P value < 0.05 was considered significant.

The following terms require to be defined-

Major accident (Effiong 1993): Any accident that resulted in damage to the vehicle beyond repairs or death to person(s) or livestock and

Minor accident (Effiong 1993): Damage to the vehicle but without loss of lives.

## Results

A total of 400 of 410 drivers (97.6%) coverage) in all the motor parks were examined. The other 10 were either on sick leave or were in transit. Majority of the drivers (n = 300, 75%) were minibus drivers. All the drivers were males.

The age range was 25 to 62 years with mean of 37.8 years (SD ± 9.1). Three hundred and fifty (87.5%) drivers were less than 50 years old.

Majority (98.2%) of the drivers have had some form of formal education and could read and write.

Twelve drivers (3%) were driving without drivers’ license. It was observed that 15% of the drivers obtained their license before the stipulated age of 18 years (Effiong 1993). The lowest age at which driving license was obtained was 11 years.

Up to 80% of the drivers underwent driving test before obtaining license though only 65% (n = 260) attended driving school.

Only 120 (30.9%) of the licensed drivers had their eyes tested prior to issuance of driving license.

**Figure 1:**
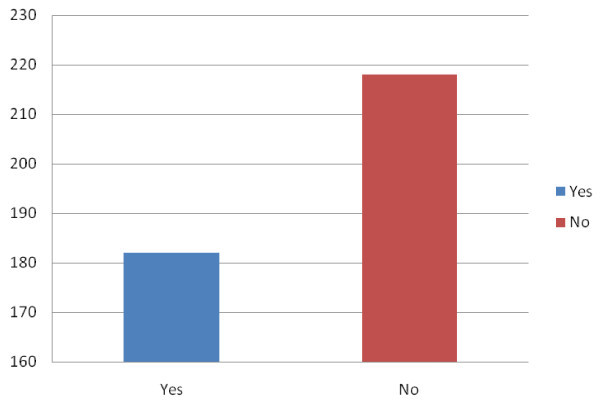
**Drivers involved in Road traffic accidents in Port Harcourt, Nigeria.**

Up to 45.5% (n = 182) drivers had been involved in RTAs. Figure 1.

The majority (62.5%) of these were between the ages of 30–49 years.

**Figure 2:**
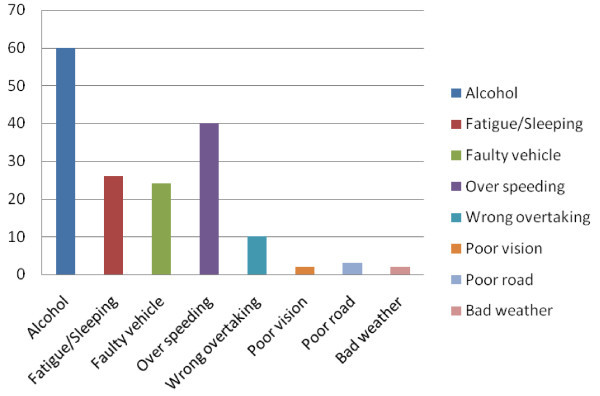
**Causes of Road Traffic Accidents in 182 of 400 drivers in Port Harcourt, Nigeria.**

The commonest causes of accidents were alcohol intoxication and driver fatigue. Figure 2.

Depressants used by the drivers while on duty include alcohol, kola nut and cigarette. While 180 drivers (40.4%) consumed alcoholic drinks alone, 110 drivers (24.7%) chewed kola nut alone, 26 drivers (5.8%), smoked cigarettes alone while 10 took bitter cola (2.2%). Only 50(12.5%) did not take any. Others took various combinations of these depressants.

By (WHO 1984) definition, most of the drivers (n = 393, 98.2%) had good vision while only 7(1.8%) had impaired vision.

The commonest symptom was blurring of vision (n = 50, 32.2%), followed by growth in the eye (n = 30, 19.4%), Itching (n = 20, 12.9%), redness (n = 20, 12.9%) and foreign body sensation (n = 20, 12.9%). About sixty (38.7%) drivers had more than one symptom.

Pterygium and presbyopia were the commonest ocular findings on examination, followed by cataract, glaucoma and refractive error. Table 1.

**Table 1:** **Ocular anomalies among drivers in Port Harcourt, Nigeria**

	Findings	Number	%
Lid	Chalazion	4	1.5
Globe	Squint	1	0.4
Conjunctiva	Conjunctivitis	15	5.7
	Pterygium	70	26.7
Cornea	Central opacity	1	0.4
Anterior Chamber	Shallow		0.4
Pupils	Miosed	1	0.4
	Synechiae	5	1.9
Lens	Cataract	37	14.1
Fundus	Glaucomatous cupping	30	11.5
	Optic atrophy	1	0.4
	Macular hole	5	1.9
	Chorioretinal scar	9	3.4
Refractive error	Myopia =20	22	8.4
	Hypermetropia =2		
Presbyopia		60	22.9
Total		262	100.0

Thirty two (8.0%) of the drivers were visually impaired (VI) in one eye with cataract, the leading cause of visual impairment in the better eyes of the affected drivers.

The relationship between VI and RTA was however not statistically significant (p = 0.46, χ ^2^ = 0.3, RR = 0.62)

None of the 182 drivers (45.5%) involved in RTAs were monocularly blind.

**Figure 3:**
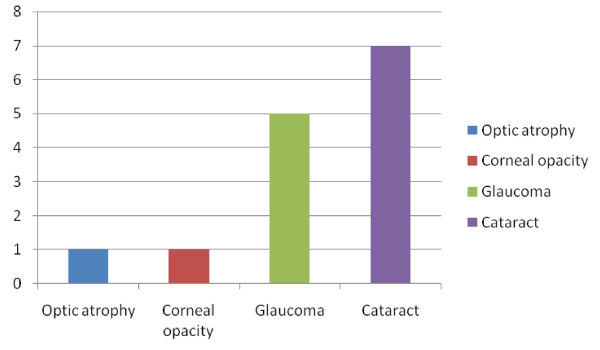
**Causes of monocular blindness among 14 of 400 drivers in Port Harcourt, Nigeria.**

Another fourteen (3.5%) were blind in one eye out of which four (28.6%) were not perceiving light (NPL) in that eye. Of these, 50% was due to cataract, 35.7% due to glaucoma and 7.1% for both optic atrophy and corneal opacity respectively. Figure 3

Sixteen (4%) of the drivers had visual field defects of which 9(2.25%) had prior history of RTAs. However this was not statistically significant. (p > 0.05,χ^2^ = 0.78,RR = 1.25)

Eighteen drivers (4.5%) were color defective and 10 of them have had a past history of RTA. However color vision is not significantly associated with RTAs in this study (p = 0.4,χ ^2^ = 0.77, RR = 1.23)

## Discussion

The resulting associated economic losses as a result of poor driving are in the region of billions (Road safety Nigeria (homepage on the internet) (Cited 2008 Dec. 10) 2013). Poor driving is also related to inability to read and write which was found in some drivers in this study (1.22%).This will make it difficult for them to read and understand street signs. It had been shown that higher educational attainment brings about knowledge of the traffic codes with resultant improvement in safety (Adogu & IIika 2006).

### Driving history

Majority (80%) underwent driving tests before being licensed. This is however lower than an Ibadan study (Nwosu 1989) (97.4%). That study however focused on government drivers in which possession of a driving license is a prerequisite. It is however important to note that 15% of the drivers in our study obtained their license before the stipulated age of 18 years (Federal Road Safety Commission 1990). This is lower than the 22.8% obtained in a similar study in Enugu (Effiong 1993). This can be attributed to the strict measures being implemented by the Federal Road Safety Commission in recent times.

It was observed that only 120 drivers (30.9%) had their eyes tested prior to issuance of driving license. This is better than a similar study carried out earlier in Nigeria (Erikitola 1998) (24.1%) probably due to subsequent increased awareness of road safety requirements since then.

The prevalence of road traffic accidents in this study was 45.5% for both “major” and “minor” accidents (Effiong 1993). This prevalence is similar to that obtained by Effiong (Effiong 1993) (43.6%) and higher than the survey in Ibadan (Nwosu 1989) in which only 3.5% admitted to being involved in RTA. This marked disparity can be attributed to the differences in study population in that the present study was on commercial drivers while the lbadan study was on government drivers who are generally more careful and in comparison probably spend less time on the road. Majority of the drivers involved in RTA in this study were between 30–49 years (62.5%) similar to other studies (Effiong 1993; Abraham 2007). Causes of accidents showed that human factor ranked highest (85%) with alcohol intake, fatigue / sleeping on wheels, excessive speeding, wrong overtaking and poor vision as the commonest causes in that order. The WHO has also identified drunken driving, excessive speeding and lack of seat belt use as major risk factors to RTA (Road safety Nigeria (homepage on the internet) (Cited 2008 Dec. 10) 2013). A Kenyan study showed human factors accounted for 85.5% of RTAs (Odero et al. 2003) as opposed to another Nigerian study by Abraham (Abraham 2007) in which human factor accounted for only 27.7%. This difference may be attributed to her slightly smaller sample size (291 drivers) and other factors such as more stringent conditions for obtaining driving license.

A large number of drivers admitted to ingestion of alcohol (45%) while on duty in our study in addition to other depressants.

Alcohol depresses the central nervous system with subsequent release of inhibition thus causing the driver to overestimate his ability while underestimating his deficiencies. Impairment by alcohol is an important factor influencing both the risk of RTA as well as the severity of the injuries that result from the RTA (Heng et al. 2006). A study carried out in the United States of America provided the basis for the international levels of blood-alcohol (BAC) set in many countries at 0.08 g/dl (Facts: Road Safety- alcohol Home page on the internet No date Cited 2007 August 28 (http://www.who.int/violence-injury-prevention.html). However more recent analyses suggest that the risks associated with these BAC levels are higher than originally thought. This has led many countries to reduce legal BAC limits to 0.05 g/dl. BAC is not yet routinely being measured in Nigeria however.

A quarter of the drivers took kola nuts which contain caffeine to control fatigue while on duty. Fatigue accounted for 28% of all causes of road traffic accident which is close to a United Kingdom study of 20% (The Royal Society for the Prevention of Accidents 2001).

### Prevalence of visual impairment (VI)

Even though VA is the only visual parameter currently measured by FRSC (Agunloye 1990b), it was noted that it was not checked in most cases before drivers were issued their driver’s license. Seven out of the 400 drivers (1.8%) were VI, similar to the findings by Effiong (Effiong 1993) (1.6%), Erikitola (Erikitola 1998) (1.7%) and Abraham (Abraham 2007) (1.7%).

But lower than the Ibadan study (Nwosu 1989) with a prevalence of 3.1%. Most of the drivers with VI were in the age group 50–69 (71.4%). As people age, visual functions deteriorate due to increase in the incidences of age related ocular conditions such as cataract, macular degeneration and open angle glaucoma.

In this study, association between VI and RTA was not statistically significant ( p > 0.46) which is similar to the findings by (Erikitola 1998) and (Cashell 1986). It was interesting to note that only 2 out of the 182 drivers (1.1%) who had been involved in road traffic accident in this study admitted to visual problems as a cause of RTA. However other workers (Nwosu 1989; Effiong 1993; Oladehinde et al. 2007) found the association between VI and RTA to be statistically significant. Owsley and Mcgwin (North 1985) in their study found no strong association between VI and unsafe driving.

### Prevalence of visual abnormalities

The causes of monocular blindness include cataract (7) 50% of the cases, glaucoma (5) 35%, optic atrophy (1) and corneal opacity (1), each contributing 7.1%. Though these 14 have not been involved in any RTA. Seven of the drivers between the ages of 30–49 attributed eye injuries (not from RTAs) as the cause of their blindness. Eye injuries are an important cause of monocular visual loss in Nigeria (Owsley & Mcgwin 1999). Trauma has also been identified as the commonest cause of monocular blindness in similar studies (Nwosu 1989; Effiong 1993; Abraham 2007). However of all the 182 drivers who have been involved in RTAs in this study, none were monocularly blind. However a study found that one-eyed drivers cause dangerous accidents at intersections three times higher than normal drivers (Federal Republic of Nigeria official Gazette 2004). This was not the case in our study.

In this study, there was no significant association between visual field defects and RTA. This agrees with other studies done in Nigeria (Nwosu 1989; Effiong 1993; Erikitola 1998; Abraham 2007) though the confrontation method was used; however Oladehinde et al (Oladehinde et al. 2007) who also used automated perimetry like in our study to evaluate similarly reported that visual field anomalies were not statistically significantly associated. Majority of the visual field defects were due to cataract and glaucoma which is similar to the Ibadan study (Nwosu 1989). Some workers outside Nigeria however found significant association between visual field defect and RTA (Cashell 1986; Burg 1975). This difference could however not be explained. This does not however mean that visual fields should no longer be evaluated to acquire a driving license.

Color vision defects seen in 4.5% of the drivers in our study is lower than other Nigerian studies carried out in Enugu (Effiong 1993) (8.1%), Lagos (Erikitola 1998) (6.9%) but higher than an Ibadan (Nwosu 1989), Nigerian study (3.3%). Color defects was not found to be statistically significantly associated with RTA (P = 0.4) in our study. A study by (Cashell 1986) also found no significant correlation between color vision defect and RTA as the color blind drivers had learnt to adjust by memorizing the colors of traffic lights by their position. Color defects can be acquired following chronic tobacco use (Kanski 2003).

Regarding the testing of eyes before issuance of driving license, it was observed that only 1/3rd (n = 120, 30%) of the drivers had their eyes tested before issuance of driving license. This shows that eye tests are not routinely carried out contrary to the 2004 National Road Traffic Regulations (Adefule & Valli 1983) which requires a VA and general fitness certificate from a government hospital prior to undergoing a driving test. Eye testing should also be periodic, not a onetime test.

### Ocular findings

Of the 400 drivers examined, 155 (38.7%) had an ophthalmic complaint. Pterygium was the commonest ocular problem seen with a prevalence of 26.7% similar to Effiong (Effiong 1993 Johnson 1984); probably because these drivers are exposed to windy and dusty conditions (Nwosu 1998).

Although distance vision is more important for driving; presbyopic symptoms which accounted for 22.9% in our study could be very distressing to older commercial motor vehicle drivers who sign movement registers and other documents in Transport Companies. Abraham found a prevalence of 30.2% (Abraham 2007).

Cataract though the commonest cause of blindness in Nigeria (Kyari et al. 2009) accounted for only 14% of ocular problems seen since most of the drivers was young. This is lower than the figure of Erikitola (Erikitola 1998) (19.3%) but higher than Nwosu (Nwosu 1989) (1.7%).

Glaucoma which is the second commonest cause of blindness in Nigeria (Kyari et al. 2009) accounted for 11.5% of diseases in this study similar to other Nigerian studies 11.6% (Erikitola 1998) and 10.9% (Abraham 2007).

Refractive error (excluding presbyopia) affected less than 10% (8.4%) of the drivers similar to another Nigerian study (Erikitola 1998). Myopia accounted for 90.9%.

Appropriate medical, surgical and optical evaluation of these ophthalmic conditions in these drivers is important in order to enhance their visual performance which will directly impact positively on safety on the roads.

## Conclusion

One out of two commercial drivers has been involved in an RTA in this cohort possibly because a third of them probably never did an eye test prior to obtaining driver’s license. This shows that it is possible to obtain a professional driving license in Rivers state, Nigeria and possibly in most other parts of Nigeria without undergoing any supervised driving test. Also despite regulations, up to 15% of commercial motor vehicle drivers obtained driver’s license before the legal age of 18 years. Though less than 2% were visually impaired, it is nevertheless dangerous for road safety for commercial motor vehicle drivers to operate their vehicles with such vision. A third of the drivers could have their vision improved with spectacles but are not wearing them. Intake of depressants should be discouraged by drivers rather emphasizing on adequate rest.

Periodic comprehensive eye testing which should include automated visual field testing and certification should be obtained from government hospitals by trained ophthalmic medical personnel prior to issuance and renewal of driving license as provided in the 2004 National Road Traffic Regulations and enforced by the appropriate authorities.
